# Renal Affections: Their Diagnosis and Pathology

**Published:** 1850-12

**Authors:** 


*Renal Affections: their Diagnosis and Pathology.* By Charles Frick, M. D. Philadelphia, Lea & Blanchard, 1850.

Dr. Frick here presents to the investigator of pathologic urine a little manual, admirably adapted to smooth away many difficulties which beset the student of this department. The work is progressive in its arrangement, leading the reader by degrees from a superficial examination to a more exact and rigid analysis of this secretion, which, in its various conditions, is now engaging so largely, the attention of pathologists and organic chemists. The work contains a large amount of original observations, and is illustrated with numerous wood cuts, which in the majority of instances have been copied by the author from the field of the microscope. We commend it heartily to the student of urinology.

